# Loneliness during the COVID-19 Pandemic in Patients with Burning Mouth Syndrome: A Multicentric Case-Control Italian Study

**DOI:** 10.1155/2023/6666741

**Published:** 2023-11-08

**Authors:** Daniela Adamo, Giulia Ottaviani, Federica Canfora, Stefania Leuci, Noemi Coppola, Giuseppe Pecoraro, Katia Rupel, Magdalena Theodora Bogdan Preda, Veronica Vello, Umberto Albert, Margherita Gobbo, Luca Guarda-Nardini, Amerigo Giudice, Elena Calabria, Massimo Aria, Luca D'Aniello, Matteo Biasotto, Michele Davide Mignogna

**Affiliations:** ^1^Department of Neuroscience, Reproductive Sciences and Dentistry, University of Naples Federico II, 5 Via Pansini, 80131 Naples, Italy; ^2^Department of Surgical, Medical and Health Sciences, University of Trieste, 447 Strada di Fiume, 34149 Trieste, Italy; ^3^Unit of Oral and Maxillofacial Surgery, Ca' Foncello Hospital, Treviso, Italy; ^4^Dentistry Unit, Department of Health Sciences, University of Catanzaro “Magna Graecia”, 88100 Catanzaro, Italy; ^5^Department of Economics and Statistics, University of Naples Federico II, 21 Via Cintia, 80126 Naples, Italy; ^6^Department of Social Sciences, University of Naples Federico II, 1 Vico Monte della Pietà, 80138 Naples, Italy

## Abstract

**Background:**

Lockdown restrictions during the COVID-19 pandemic exerted a strong impact on people's quality of life and increased loneliness. This study evaluates the effect of the pandemic on loneliness in patients with burning mouth syndrome (BMS) compared with the general population.

**Methods:**

100 BMS patients versus 100 healthy controls (HC) were recruited in five Italian centers. The 12-Item General Health Questionnaire (GHQ-12), the Depression Anxiety Stress Scales-21 (DASS-21), the Insomnia Severity Index (ISI), the short-form UCLA Loneliness Scale-8 (ULS-8), the Multidimensional Scale of Perceived Social Support (MSPSS), and the Suicidal Ideation Attribute Scale (SIDAS) were administered.

**Results:**

BMS patients and HC showed high scores (16 [14-20.25] and 16 [14-18]) in the ULS-8. Statistically significant differences have been found considering the BMS patients lived with fewer relatives during the lockdown compared with the HC (2 [2-3] and 3 [2-4]; *p*: 0.012) with a lower level of satisfaction in relationships with relatives (4 [1.75-5] and 5 [4-5]; *p* < 0.001) and also in the DASS-21 total scores between the BMS patients and HC (16 [10-24.2] and 10 [4-17]; *p* < 0.001). The multivariate logistic regression revealed that age, education, DASS-21, and MSPSS were the most predictive variables and could explain 34.68% of the variance in the ULS-8 score (*p* < 0.001) in the BMS group. However, only the DASS-21 was significant in the HC group, explaining 10.11% of the variance of the ULS-8 (*p*: 0.033).

**Conclusions:**

Both the patients and controls experienced deep loneliness during the pandemic. However, in the BMS group, loneliness was significantly correlated with age, a higher level of education and stress, and a lower level of satisfaction in relationships with relatives and social support perceived compared with the controls.

## 1. Background

The corona virus disease 2019 (COVID-19) pandemic is one of the most important healthcare and societal challenges to have emerged in this century. Italy was the first country to be struck by COVID-19 in Europe and the first country to impose a national lockdown as an attempt to prevent the spreading of the coronavirus, confining over 60 million people at home [1, 2].

The fear of contagion, the economic crisis, the social and physical distancing due to the quarantine measures [3], and the travel restrictions especially during the lockdown but also in the postlockdown pandemic contributed to increase the level of stress, anxiety, and depression related to health [4], intensifying a pervasive subjective feeling of loneliness and social isolation especially in elderly and vulnerable people suffering from chronic pain [5].

Previous researches have suggested that high stress situations such as wars, terrorist attacks, and natural disasters adversely affect the severity of symptoms and psychological comorbidities in chronic pain patients, resulting in an exacerbation of the disease with an impairment of the patient's quality of life [5, 6]. In particular, social isolation and subjective loneliness represent important risk factors for mental health deterioration, a fact which has been ignored for a long time [7, 8]. However, considerable evidence has documented an increased perception of loneliness during the pandemic outbreak [9, 10], which in turn was identified as a risk factor for mood disorders, cardiovascular diseases, and systemic comorbidities, resulting in premature mortality [11] but also increasing the suicidal ideation prevalence [12].

Currently, there is no consensus about the meaning of “loneliness,” the most commonly used definition being “an emotionally unpleasant experience in which the individual feels a discrepancy between the interpersonal relationships that she/he has and those that she/he perceives” [13]. Two distinct types of loneliness have been described, with a distinction between emotional and social loneliness, often overlapping. Emotional loneliness is defined as “resulting from the lack of a close, intimate attachment with others” and social loneliness as “resulting from the lack of a network of social relationships in which the person is part of a group of friends who share common interests and activities” [14]. During the pandemic, social isolation contributed to physical loneliness, perceived as the lack of any physical contact, increasing in turn emotional and social loneliness mainly in the elderly population [15].

Burning mouth syndrome (BMS) is a chronic, idiopathic pain disorder characterized by a burning/dysesthetic sensation in the oral cavity lasting for more than three months in the absence of any local or systemic pathological changes [16]. The worldwide prevalence of BMS is 1.73% in the general population with the highest prevalence in middle age or older female [17]. Several additional oral and extraoral symptoms have been reported, with burning negatively affecting the psychological profile and quality of life of such patients [18]. Mood disorders, sleep disturbance, and cognitive impairment frequently overlap with BMS, contributing to the aggravation of the disease [19–21].

During the pandemic, loneliness was regarded as one of the most important factors affecting individual psychological well-being [11]. Therefore, in this context, a high level of perceived loneliness may be considered not only as a trigger for the onset of the disease but also as a condition liable to amplify the symptomatology and the mood disorders also in patients already affected by BMS.

Several studies have found an increased level of loneliness, anxiety, depression, and stress, as well as a rise in suicide rates during the pandemic [22, 23]. Others, in contrast, have revealed no significant changes in level of loneliness [24]. However, to our knowledge, no research has examined this dimension in relation to BMS.

Therefore, the present study has been carried out in order to analyze the effect of the COVID-19 pandemic and lockdown restrictions in a group of patients suffering from BMS compared with a group of healthy controls (HC).

The primary endpoint of the study has been to evaluate the following, in a group of patients suffering from BMS compared with an equal number of healthy controls (HC):

- Levels of loneliness, perceived social support, and satisfaction in relationships
- The utilization of social media and healthcare services
- Stress, anxiety, depression, and sleep disturbance

The secondary endpoint has been to analyze the following:

- The predictors of the dimension of loneliness in both groups, taking into account the sociodemographic profile, risk factors, systemic comorbidities, drug consumption, pain, psychological factors, and satisfaction in relationships
- Information about the onset and worsening of BMS in relation with COVID-19 pandemic and the changes in treatment after the COVID-19 pandemic

Our hypothesis was that the COVID-19 pandemic would cause a higher perception of loneliness in the BMS patients compared with the healthy subjects, with a subsequent worsening of the psychological profile and symptomatology.

## 2. Methods

### 2.1. Study Design and Participants

This is a multicentric observational cross-sectional study which was carried out between June 2022 and January 2023, involving patients from the Oral Medicine and Pathology Unit of the University of Trieste, the Oral Medicine Department of University of Naples “Federico II”, the Unit of Oral Maxillofacial Surgery of Treviso (Ca' Foncello Hospital), the Departmental Structure of Odontostomatology (ASFO, Pordenone), and the Oral Surgery and Pathology Unit of the University Magna Graecia of Catanzaro. The recruitment was performed in accordance with the ethical principles of the World Medical Association Declaration of Helsinki after approval by the Ethical Committee of the University (Approval Number: 251/19: February 20, 2019) and in accordance with the Strengthening of the Reporting of Observational Studies in Epidemiology (STROBE) guidelines for observational studies [25].

The sample size, equal to 100 patients for each group (the BMS patients and HC), was calculated to obtain a power test value (1-beta) at no less than 99%, associated with a significance of no more than 1%. This sample size was obtained using the effect size value equal to 1.28, measured in a previously published research study [20]. The calculations were computed using the GPower software v. 3.1.9 [26].

**Figure 1:**
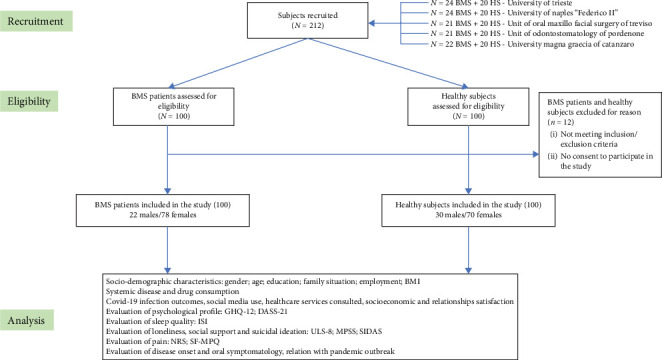
Flow chart of the study. BMS: burning mouth syndrome; BMI: body mass index; GHQ-12: 12-Item General Health Questionnaire; DASS-21: Depression Anxiety Stress Scales-21; ISI: Insomnia Severity Index; ULS-8: UCLA Loneliness Scale-8; MSPSS: Multidimensional Scale of Perceived Social Support; SIDAS: Suicidal Ideation Attribute Scale; NRS: Numeric Rating Scale; SF-MPQ: short-form McGill Pain Questionnaire.

All diagnosed patients with BMS and healthy patients were invited to participate in the study, and a written informed consent was obtained before the enrolment, taking into account the inclusion and exclusion criteria of the study. The healthy subjects were enrolled during the same recruitment period as the BMS patients and selected from patients attending our hospital facility for other medical or nonmedical reasons. Both BMS patients and healthy subjects have been enrolled in the study after obtaining the consent. The recruitment planning and development has been described in the flow chart of the study (Figure 1).

### 2.2. Assessment of BMS Patients and HC

The inclusion criteria for BMS patients were as follows:

- An intraoral burning or dysesthetic sensation, recurring daily for more than two hours per day and lasting for more than three months, in accordance with the definition of the International Classification of Orofacial Pain [16], without evident causative lesions on clinical examination
- No alterations of routine blood tests (blood count, blood glucose levels, glycated haemoglobin, serum iron, ferritin, and transferrin)

The inclusion criteria for healthy controls were as follows:

- Subjects without any lesion of the oral mucosa
- Subjects without a history of BMS
- Subjects with normal blood test findings (blood count, blood glucose levels, glycated haemoglobin, serum iron, ferritin, and transferrin)
- Subjects who had not undergone treatment with psychotropic drugs

The exclusion criteria for the healthy control and BMS patients were as follows:

- Patients aged lower than 18
- Subjects with a psychiatric disorder or a neurological or organic brain disorder
- Patients having a history of alcohol or substance abuse
- Patients unable to understand or complete the questionnaires

### 2.3. Anamnestic and Clinical Data

The oral medicine specialists, DA (Naples) and GO (Trieste), MG (Treviso) and MB (Pordenone), and AG (Catanzaro), carried out the intraoral and extraoral standardized screening of each patient in order to obtain consistent results. Specific anamnestic data were acquired during the first consultation, namely, the sociodemographic profile (gender, age, education profile, family status, and employment status), body mass index (BMI), systemic disease history, and drug intake.

### 2.4. COVID-19 Infection Outcomes, Social Media Use, Healthcare Services Consulted, and Socioeconomic and Relationship Satisfaction

The COVID-19 infection history was assessed through written questions enquiring about any previous infections, any periods of quarantine after COVID-19 infection, and any periods of quarantine on account of any COVID-19 infection of a relative. Information about time spent on the Internet and the use of social media (instant messaging, social networks, searches for information, entertainment, shopping online, booking of travel/social events, financial services blogs/debating online/forums, and education/learning services) was collected. The score was calculated in accordance with the following scale: never, 0; rarely, 1; sometimes, 2; often, 3; and always, 4.

Information about healthcare services consulted during the pandemic and about financial gratification after the pandemic was recorded.

Socioeconomic satisfaction and living place satisfaction was evaluated using a score ranging from 0 to 6 with higher scores representing a greater satisfaction. Social isolation or social loneliness was evaluated with questions regarding the number of relatives at home and the level of satisfaction in the relationships with such relatives. The level of relationship satisfaction was assessed with scores ranging from 0 to 6 with higher scores representing a greater satisfaction.

The following self-administered questionnaires were used to evaluate the corresponding clinical parameters, and patients were asked to answer with reference to the COVID-19 lockdowns.

### 2.5. Psychological Profile Assessment

- The 12-Item General Health Questionnaire (GHQ-12), Italian validated version [27], was used to detect minor (nonpsychotic) psychiatric disorders
- The Italian version of the Depression Anxiety Stress Scales-21 (DASS-21) [28] is a self-reported measure able to assess, using 21 items scored in a three-factor oblique model, the characteristics of stress, anxiety, and depression

### 2.6. Sleep Quality

The self-reported questionnaire Insomnia Severity Index (ISI) [29] was used to assess the patient's perception of insomnia, taking into account 7 items evaluating the daytime consequences of insomnia and any subsequent distress.

### 2.7. Loneliness, Social Support, and Suicidal Ideation

- The short-form UCLA Loneliness Scale- (ULS-8) [30] is an 8 item self-reported questionnaire used to evaluate the perceived social isolation
- The Multidimensional Scale of Perceived Social Support (MSPSS) [31] is a 12-item measure of the perceived adequacy of social support
- Suicidal ideation was evaluated using a single-item Suicidal Ideation Attribute Scale (SIDAS) [32]

Cronbach's alpha index was performed to assess the internal consistency and global reliability of the GHQ-12, DASS-21, ISI, ULS-8, MPSS, and SIDAS. This measure allowed us to examine the consistency of each item with the overall scale as well as the reliability of the scale.

All the scales demonstrated a good reliability with the following Cronbach's alpha index scores: GHQ-12: 0.648, DASS-21: 0.947, ISI: 0.88, ULS-8: 0.654, and MSPSS: 0.945.

### 2.8. Disease Onset, Pain Assessment, Symptomatology, and Relation with Pandemic Outbreak

The anamnestic data for the BMS patients were analyzed, and the timing of the onset of symptoms was recorded in order to calculate the diagnostic delay and the number and specialty of the doctors consulted before the diagnosis. The evaluation of the disease was completed with questions about the disease onset, specifically whether it was antecedent or subsequent to the outbreak of the pandemic and whether there was any worsening of the disease during that period. Information regarding the treatment was recorded in relation to any psychotropic drugs or palmitoylethanolamide (PEA) taken. The evaluation of the pattern of symptoms over time was addressed to any daily variation (the timing of any worsening) and their latency during the nighttime. Moreover, all the oral symptoms reported and their localisation were recorded and estimated through the Numeric Rating Scale (NRS), scored from 0 to 10, and the short-form McGill Pain Questionnaire (SF-MPQ) characterized by 11 items measuring the sensory qualities of the pain experience [33, 34].

The questionnaires' design and instructions have been uploaded in the Supplementary file (available here).

### 2.9. Statistical Analysis

The R software (v. 4.1.2; Team RCore, 2016) [26] was used to carry out the statistical analyses. The sociodemographic and clinical characteristics were summarized by calculating descriptive statistics, comprising means, standard deviations (SDs), medians, and interquartile ranges (IQRs). Fisher's exact test was employed to evaluate any significant differences in the frequencies of the sociodemographic characteristics, body mass index, systematic diseases, drug consumption, COVID-19 infection, healthcare services, DASS-2, and ISI between the BMS patients and healthy subjects. The Mann–Whitney *U*-test, a nonparametric statistical test, was employed to assess differences among the clinical parameters, social media use, socioeconomic satisfaction, satisfaction about the place of residence, social loneliness, GHQ subitems and total score, DASS-21 total score, ISI total score, and the subitems and total score of the ULS-8, MPSS, and SIDAS. This nonparametric test was chosen based on the assessment of variable normality through the Shapiro-Wilk test indicating that each variable exhibited a nonnormal distribution. Then, a multivariate linear regression analyses were conducted using all possible variables identified as predictors. In detail, a sequential regression model analysis was performed where the predictors were added one by one to obtain unadjusted coefficient estimations. In a final step, we performed a full model analysis considering all the predictors simultaneously to estimate the adjusted coefficients and recorded the adjusted *R*^2^. The adjusted *R*^2^ was used to measure the overall goodness of fit of each model, taking into account the number of variables included in the model. In all the steps, we reported the standard errors of the model coefficients, which provide a measure of the statistical precision of the inference estimation of the model parameters.

The gender, age, years of education, BMI, number of relatives in the home, satisfaction in relationships, COVID-19 infection, primary care physician consultations, hypercholesterolemia, use of proton pump inhibitors, DASS-21, GHQ12, and MSPSS were considered for the BMS patients and HC. The NRS and SF-MPQ were also considered as predictors in the BMS patients.

## 3. Results

A total of 200 participants were enrolled, 100 BMS-affected patients and 100 HC. Their sociodemographic profile and body mass index (BMI) are summarized in Table 1.

In the overall sample, consisting of the BMS patients and HC, women accounted for 78% and 70%, respectively, with no statistically significant difference between the two groups (*p* value: 0.259). Furthermore, there was no difference in the mean age (*p* value: 0.957) and BMI (*p* value: 0.283).

Both the BMS and healthy subject groups resulted homogeneous considering the gender, ages, family situation, and BMI. Statistically significant differences were highlighted in relation to education and employment. The BMS patients presented a lower education (11.1 ± 4.66 years) compared to the HC (13.5 ± 3.42 years) (*p* value: <0.001). A lower percentage of the BMS patients were employed (BMS: 39%, HC: 56%), while a higher number were unemployed (BMS: 33%, HC: 21%) or retired (BMS: 28%, HC: 23%) (*p* value: 0.045).

Table 2 shows the prevalence of systemic diseases and the drug consumption between the groups. In general, the BMS patients were more frequently affected by systemic diseases (54%) than the HC (41%) (*p* value: 0.089). It is noteworthy that a higher percentage of the BMS patients suffered from hypercholesterolemia (14%) compared to the HCs (2%) (*p* value: 0.003). A significant difference was observed with respect to the drug consumption: 44% of the BMS patients took medications, compared with 24% among the HC (*p* value: 0.004). This difference was noticed particularly in relation to the assumption of statins and proton pump inhibitors, which were prevalent in the BMS patients (*p* value: 0.003 for both items).

Table 3 shows the prevalence of COVID-19 infection, social media use, access to healthcare services, socioeconomic satisfaction, and feeling of social loneliness during the COVID-19 pandemic between the groups.

A statistically significant difference was found in the use of education/learning services between the BMS patients and HC (0 [0-2] and 1 [0-3], respectively; *p* value: 0.005).

The BMS patients consulted more frequently primary care physicians (57%) compared to the HC (32%) (*p* value: 0.001). The BMS patients lived with a lower number of relatives (*p* value: 0.012), and their satisfaction with respect to their family members was significantly lower than that of the HC (*p* value: <0.001).

An analysis of the subscores and total score of the GHQ-12, DASS-21, and ISI in the BMS patients and HC is presented in Table 4.

As regards the GHQ-12, the BMS patients felt less skilled in formulating decisions (GHQ-4; *p* value: 0.001) and unhappier considering all their circumstances (GHQ-12; *p* value: <0.001). No significant differences were detected in their capability to concentrate (GHQ-1; *p* value: 0.009), in the sensation of sleep loss due to worry (GHQ-2; *p* value: 0.009), in the feeling of playing a useful role in events (GHQ-3; *p* value: 0.160), and in the sensation of being constantly overwhelmed and stressed (GHQ-5; *p* value: 0.332).

The DASS-21 total scores showed a higher median score in the BMS patients (16; 10-24.2) than in the HC (10; 4-17) (*p* value: <0.001). Specifically, a high level of the subscore for stress was found in the BMS patients compared with the HC (*p* value: <0.001). It is noteworthy that the BMS patients reported higher levels of moderate and extremely severe stress (22% and 4%, respectively) in comparison with the HC (5% and 0%, respectively).

The ISI revealed that the two groups were similar as regards the evaluation of insomnia (*p* value: 0.634), as can be observed in the median of the ISI total score (*p* value: 0.265).

Table 5 shows the analysis of the subscores and total score of the ULS-8, MSPSS-12, and SIDAS.

The median and IQR of the total score of the ULS-8 were similarly high in both the BMS patients and HC (16 [14-20.25] and 16[14-17.25]; *p* value: 0.017). The ULS-8 revealed that the BMS patients felt unhappier in lockdown than the HC (ULS-7; *p* value: 0.004). The sensation of lacking companionship (ULS-1; *p* value: 0.078), the absence of someone who they could turn to (ULS-2; *p* value: 0.144), the feeling of being an outgoing person (ULS-3; *p* value: 0.078) and being left out (ULS-4; *p* value: 0.575), the perception of isolation from others (ULS-5; *p* value: 0.170), the possibility of companionship in every moment (ULS-6; *p* value: 0.968), and the sensation of being isolated among people (ULS-8; *p* value: 0.292) were similar in the two groups.

The BMS patients and HC had someone to contact in case of need (MSPSS-1; *p* value: 0.080) and with whom they could share joys and sorrows (MSPSS-2; *p* value: 0.044), a family member who tried to help them (MSPSS-3; *p* value: 0.038) and gave them emotional help and support (MSPSS-4; *p* value: 0.452), and a special person who was able to comfort them (MSPSS-5; *p* value: 0.146). Likewise, they also had friends who tried to help them (MSPSS-6; *p* value: 0.176) and who they could count on when things went wrong (MSPSS-7; *p* value: 0.353). Both groups revealed that they could talk about their problems with their family (MSPSS-8; *p* value: 0.481), that they had friends with whom they shared joys and sorrows (MSPSS-9; *p* value: 0.795) and a special person who cared about their feelings (MSPSS-10; *p* value: 0.422), and that they had a family member who was willing to help them make decisions (MSPSS-11; *p* value: 0.315) and friends with whom they could talk about their problems (MSPSS-12; *p* value: 0.578).

The SIDAS revealed how often the BMS patients and HC had had suicidal thoughts in the last year: the median score was 0 for both groups (*p* value: 0.335).

The results in relation to the disease onset; type of treatment; number of consultations prior to the diagnosis; type and number of referrals; the intensity, quality, and pattern of the pain; and the prevalence of oral symptoms and their location in the BMS patients are reported in Table 6.

The mean onset of the disease was 30 months (median and IQR: 12-49.5 months), and the mean number of doctors consulted prior to the diagnosis of BMS was 2 (interquartile range: 1-3).

A great number of patients (63%) had presented BMS symptoms before the COVID-19 pandemic outbreak; and 27% of patients reported a worsening of the disease during the pandemic (median time of worsening: 12 months). Psychotropic drugs were used for the treatment of BMS symptoms by 62% of patients, while palmitoylethanolamide was used by 29% of patients; only 13% reported an increase in the dosage of the drugs due to a worsening of the disease. The BMS patients rated their pain as 6 according to the NRS (interquartile range: 4-8) and the SF-MPQ (interquartile range: 1-13.25).

The symptoms changed day by day in 45% of the BMS patients; 36% presented symptoms during the night. A worsening in the afternoon/evening was reported by 32% of the patients, while a lower number of patients reported a worsening in the perception of the symptoms (9%) or a stability in their symptomatology during the whole day (14%). Most of the BMS patients (89%) reported additional symptoms to the burning sensation: xerostomia (63%), dysgeusia (42%), and a subjective change in tongue color (32%) were the most common symptoms mentioned.

The results of the multiple linear regression analyses for the BMS patients and HC predicting the ULS-8 score are shown in Tables 7 and 8. The first model tested the contribution of the demographic variables to the subjective feelings of loneliness (ULS-8 which was confirmed only in the study group (*p* value: 0.024) but not in the control group (*p* value: 0.081). The addition of model 2 (which comprises the items related to the number of relatives at home and the satisfaction in relationships with relatives during the COVID-19 outbreak), of model 3 (an increased use of social media and the Internet during the pandemic), and of model 4 (the items related to the previous presence of COVID-19 infection and contact with a healthcare service primary care physician) did not result in a significant increase in the *R*^2^ value for the ULS-8 in either of the two groups. Likewise, the addition of statin intake, proton pump inhibitor intake (models 5 and 6 in the BMS patients), and hypercholesterolemia (model 7 in the BMS patients and model 5 in the HC) did not contribute to any significant increase in the *R*^2^ value for the ULS-8.

Conversely, the addition of the DASS-21 total score which is related to stress, anxiety, and depression (model 8 in the BMS patients and model 6 in the HC) significantly contributed to the ULS-8 in both groups (BMS: DR2 = 30.86, *p* value: <0.001; HC: *R*^2^ = 7.41, *p* value: <0.004).

While the addition of the GHQ-12 (general health, model 9 in the BMS patients and model 7 in the HC) was not significant for either the BMS patients or HC, the addition of the ISI and of the MSPSS (sleep and social support, models 10 and 11 in the BMS patients and models 8 and 9 in the HC) resulted in a significant increase in the *R*^2^ value of the ULS-8 only in the BMS group (ISI: DR2 = 6.86%, *p* value <0.004; MSPSS: DR2 = 18.55%, *p* value <0.001) (Tables 7 and 8).

With regard to the BMS patients only, the addition of the pain scores of the NRS and SF-MPQ (models 12 and 13, Table 7), revealed that only the quality of pain (SF-MPQ) and not the intensity of pain (NRS) contributed to the increase in the *R*^2^ value for the ULS-8 (SF-MPQ; DR2 = 5.25%; *p* value: 0.012).

In the BMS patient group, the final full model (model 14), in which all of the variables were entered simultaneously, could explain 34.68% of the variance in the total ULS-8 score (*p* value: <0.001), with the most predictive variables being age, education, the DASS-21 total score, and the MSPPS-12 total score (*p* values: 0.011, 0.046, <0.001, and <0.001, respectively) (Table 7). Instead, in the HC group, the final full model (model 10) could explain only 10.11% of the variance of the ULS-8 score (*p* value: 0.033), with only the DASS-21 total score being a significant contributing factor (*p* value: 0.004) (Table 8).

## 4. Discussion

The COVID-19 pandemic has affected and changed the lives of people around the world [35]. For some individuals, these social changes have presented opportunities (e.g., more time with the family, flexible working, and a reduced demand for travel) [36]; for others, these changes have had a substantial impact on their mental health, especially in vulnerable subjects affected by chronic pain such as BMS who have already shown high level of anxiety, depression, sleep disturbance, hypochondriac behaviors, and low stress tolerance [35, 37]. In this scenario, the physical and social distancing measures have contributed to loneliness [14, 22].

Despite loneliness emerges as a result of COVID-19 pandemic, this dimension was already known to be a significant public health issue prior to COVID-19 [38]. However, it has frequently been underestimated and has never been evaluated in patients with BMS.

Several longitudinal studies, conducted prepandemic, have shown that loneliness predicted an increase in the risk of developing mood disorders especially in patients with a chronic condition [39–41]. In addition, meta-analysis studies have found evidence that loneliness may also increase the risk of dementia, highlighting the importance of the early detection and treatment of this condition, independently of the COVID-19 pandemic [42, 43].

A recent review which has involved 34 studies has confirmed a robust increases in loneliness during the COVID-19 pandemic across gender and age groups, yielding higher continuous loneliness scores than the prepandemic assessments [10]. Moreover, a recent investigation of 58,612 invited participants has found that loneliness is strongly associated with the risk of developing signs and symptoms consistent with long COVID-19 [44].

The results of our study have suggested a high level of loneliness in the BMS patients and HC without any significant differences between the two groups. However, the BMS patients felt unhappier in lockdown compared with the HC (ULS-7). Moreover, although the pandemic offered the opportunity to have more frequent contacts with family members, improving social bonds [45], the BMS patients lived with fewer relatives, with a lower level of satisfaction in such relationships, compared with the HC, which has only contributed to increase their physical and emotional loneliness. In this context, the patients experienced sharing an enclosed space with their relatives, who generally could not perceive their pain, thereby increasing interpersonal conflicts and subsequently further isolation, as suggested by the increased subscores of the DASS-21. Indeed, a higher level of perceived stress was found in the BMS patients compared with the HC.

It is known that the pandemic and the lockdown have been considered as triggers for stressful experiences [44, 46], which in turn may have caused and exacerbated chronic pain conditions such as BMS. It is also possible to consider that the high level of perceived stress of the BMS patients may have been amplified by the reduction in the accessibility to high-quality care for pain services during the pandemic, which in turn may have increased the frequency of consultations with primary care physicians, who generally have a poor knowledge about this disease [47].

Interestingly, the predictors of loneliness were different in the BMS patients and HC. Loneliness was positively correlated with age, education, depression, anxiety, stress, and sleep disturbance and negatively correlated with perceived social support in the BMS patients but positively correlated only with anxiety, depression, and stress in the HC.

Therefore, these results suggest that older BMS patients, with a higher level of education; with higher scores for depression, anxiety, and stress; and with poorer self-perceived social support, may be at risk of developing profound loneliness. In line with current literature, older individuals may be at greater risk of social isolation, loneliness, and perceived stress [14, 48]. In this context, the prolonged activation of the hypothalamic pituitary adrenal axis and the overexpression of the proinflammatory cytokines promoting these psychological conditions have been suggested to compromise neural responses, which may directly influence the development of neurodegenerative disorders [49, 50].

The results of this study are slightly different compared with previous studies in which a younger age, being a woman, and being a student were identified as risk factors for loneliness during the pandemic [51, 52]. Conversely, in line with other studies, a higher level of social support represents a protective factor against loneliness [7].

In agreement with the literature, psychological impairment, probably exacerbated by the pandemic, was the most important predictor of loneliness in the BMS patients and HC [53–55]. Specifically, differences between the stress subitems in the DASS-21 between the two groups highlighted that during the COVID-19 pandemic, the BMS patients became more vulnerable to stress compared with the HC. For this reason, BMS patients require extra vigilance by clinicians in order to identify psychological distress at an early stage, which in time might worsen the disease over time even in patients under therapy.

In this study, in a single model, a significant correlation was found between loneliness and sleep disturbance, supporting the results of previous longitudinal studies in which a bidirectional and synergic relationship between insufficient sleep, loneliness, and social isolation has been reported [56, 57].

Social media usage has been ambiguously related to loneliness [58]. In a recent study performed in four countries, an increased time spent on Internet use during the pandemic has been reported with frequent younger users of social media feeling lonelier compared to less frequent social media users [59]. However, in middle-aged users, an increased use of social media was associated with lower levels of loneliness [60]. In this study, an increased Internet usage was found only in 37% of the BMS patients and in 38% of the HC with a short median usage time (about 2 hours/daily). The differences in the consultation of education/learning services may be explained by the higher level of education and higher percentage of employment in the HC compared with the BMS patients.

The results of the present study were in agreement with the English study of Kung and Steptoe [61] using data on 6,840 adults aged older than 50, which did not find any changes in daily Internet usage between 2018/2019 and June/July 2020, despite the increased digitalization of services during the pandemic.

Although a moderate correlation was revealed between the quality of pain and loneliness, in the final model of the regression analyses in this study, the intensity and quality of pain were not found to have significantly contributed to loneliness. However, it is possible to consider that feeling lonely, in the long term, may increase pain, as suggested by longitudinal studies that have found a bidirectional interaction between loneliness and pain catastrophizing thinking [62, 63].

Previous research has suggested that BMS may be a complication of COVID-19, considering the infection as a cause of a peripheral and central neuropathy, which in turn may promote the onset of BMS [64]. On the contrary, the results of this study have found that only 37% of patients reported the onset of the disease after the pandemic. Moreover, only 9.9% contracted the COVID-19 infection, and no patients reported the onset of the symptoms during the first month after healing. Among the BMS patients who contracted COVID-19, none reported symptoms of fatigue, brain fog, dyspnea, digestive issues, or loss of taste and smell, representative of long COVID-19.

Therefore, in line with the current literature [3, 47, 55], it is possible to consider that the stress related to the pandemic may have played a major role in the increased development and incidence of BMS rather than this resulting from any direct effect of the virus on the peripheral or central nervous system.

Additionally, the majority of the BMS patients (63%) in this study contracted the disease before the outbreak of the pandemic, and only 27% reported a worsening of the symptomatology with 13% needing to increase the dosage of any ongoing treatment. These results may be explained by the fact that most BMS patients were undergoing treatment with psychotropic drugs and antioxidants, which probably had a protective role, thereby avoiding any exacerbation of the psychological impairment and symptomatology. Moreover, clinicians kept in touch with the BMS patients throughout the pandemic by means of telehealth.

This study has certain limitations. First, considering the study's evaluation timing, we must take into account that patients were asked to complete the questionnaires with regard to the two-year pandemic time period prior to the visit. Then, although the design of the study has given a snapshot of aspects of self-perceived loneliness during the pandemic, it is not able to reveal the mechanisms underlying the increase in loneliness. Moreover, the small size of the sample, consisting predominantly of females (78%), may not be representative of the whole population, limiting generalizability. Therefore, it should be considered as an exploratory study only. Finally, the information about the COVID-19 experience, submitted to the clinicians by the patients, was self-reported with a possible consequent lack of validity.

## 5. Conclusions

The COVID-19 pandemic and accompanying lockdown restrictions adversely affected the well-being of individuals, increasing loneliness in the BMS patients but also in the HC. A higher level of perceived stress and a poorer satisfaction in relationships with their relatives were found in vulnerable subjects affected by BMS more frequently in comparison with the HC. Loneliness positively correlated with age, education, depression, anxiety, and stress and negatively correlated with perceived social support in the BMS patients while it was positively correlated only with anxiety, depression, and stress in the HC. Therefore, the older BMS patients, with a higher level of education; higher scores of depression, anxiety, and stress; and poorer self-perceived social support may be at risk of developing a profound loneliness.

**Figure 2:**
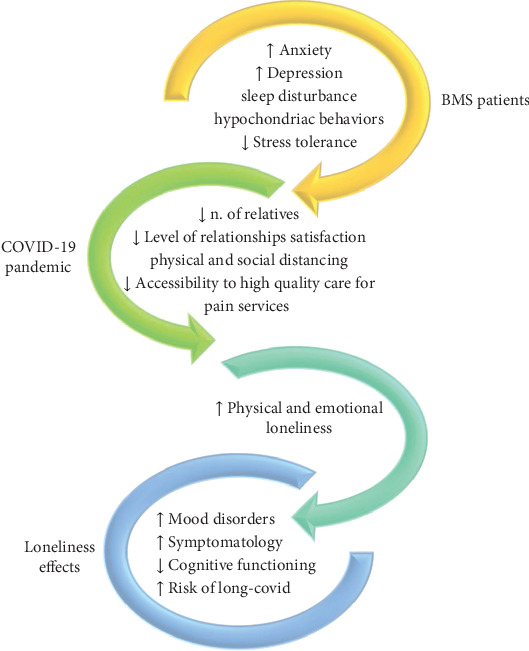
Loneliness in BMS patients related to COVID-19 pandemic.

Loneliness, social isolation, poor social and family support, and high perceived stress, when not identified and managed, may aggravate the disease and mood disorders and may represent risk factors for cognitive decline also in BMS patients under treatment. Consequently, loneliness should be considered as a new dimension that could potentially aggravate the disease (Figure 2).

These results may have some implications for clinicians, who should screen loneliness as an adjuvant tool in the assessment of BMS especially in older patients who generally already suffer from worries about the future, feelings of insecurity, negative thoughts, and sadness, which may contribute to a self-perceived loneliness.

When stressful life events happen, such as the COVID-19 pandemic, BMS patients are in greater need of psychological support in addition to physical care. Therefore, despite the difficulty in keeping in touch with patients during challenging conditions, clinicians should adopt new skills and technologies in the promotion of alternative means of communication using telehealth to replace actual face-to-face interaction in order to maintain satisfactory communication levels with patients and to identify, address, and treat loneliness and psychological distress at an earlier stage, thereby avoiding any worsening of the disease.

Individuals experiencing loneliness tend to have higher rates of healthcare utilization, seeking medical attention and interventions more frequently. Understanding and addressing loneliness in the elderly population with BMS and/or other chronic pain conditions is imperative for enhancing their overall well-being and optimizing healthcare outcomes. The significance of this issue extends beyond the confines of the COVID-19 pandemic, emphasizing the necessity of ongoing research and targeted interventions to mitigate loneliness and improve the lives of the elderly population dealing with chronic pain.

## Figures and Tables

**Table 1:** Sociodemographic profile and BMI in 100 BMS patients and in 100 healthy controls.

Demographic variables	BMS	HC	*p* value
Gender	Frequency (%)	Frequency (%)	
Male	22 (22)	30 (30)	0.259
Female	78 (78)	70 (70)	
Age (in years)	Mean ± SD 60.8 ± 13.3	Mean ± SD 60.7 ± 6.51	0.957
Education (in years)	Mean ± SD 11.1 ± 4.66	Mean ± SD 13.5 ± 3.42	<0.001⁣^∗∗^
Family situation	Frequency (%)	Frequency (%)	
Single	17 (17)	5 (5)	0.054
Married	67 (67)	79 (79)	
Divorced	7 (7)	8 (8)	
Widowed	9 (9)	8 (8)	
Employment	Frequency (%)	Frequency (%)	
Employed	39 (39)	56 (56)	0.045⁣^∗^
Unemployed	33 (33)	21 (21)	
Retired	28 (28)	23 (23)	
Body mass index (kg/m^2^)	Frequency (%)	Frequency (%)	
BMI < 18.5	5 (5)	2 (2)	0.283
BMI: 18.5-24.9 normal	46 (46)	47 (47)	
BMI: 25.0-29.9 overweight	29 (29)	41 (41)	
BMI: 30-34 class I obesity	16 (16)	10 (10)	
BMI: 35-39.99 class II obesity	3 (3)	0 (0)	
BMI > 40 class III obesity	1 (1)	0 (0)	
BMI	Mean ± SD 25.9 ± 4.76	Mean ± SD 25.2 ± 3.75	

The significant difference between means was measured by the Student *t*-test. ⁣^∗^Significance 0.01 < *p* ≤ 0.05. ⁣^∗∗^Significance *p* ≤ 0.01. The significant difference between percentages was measured by the Pearson chi-square test. ⁣^∗^Significance 0.01 < *p* ≤ 0.05. ⁣^∗∗^Significance *p* ≤ 0.01. Abbreviations: BMS: burning mouth syndrome; HC: healthy controls; BMI: body mass index.

**Table 2:** Prevalence of systemic diseases and drug consumption in 100 BMS patients and 100 healthy controls.

	BMS	HC	*p* value
	Frequency (%)	Frequency (%)	
Systemic diseases			
Yes	54 (54)	41 (41)	0.089
No	46 (46)	59 (59)	
Hypertension	23 (23)	15 (15)	0.207
Hypercholesterolemia	14 (14)	2 (2)	0.003⁣^∗∗^
Other cardiovascular diseases	7 (7)	2 (2)	1.000
Hypothyroidism	11 (11)	4 (4)	0.105
Hyperthyroidism	0 (0)	1 (1)	1.000
Endocrine disease	6 (6)	2 (2)	0.279
Gastroesophageal reflux disease	13 (13)	2 (2)	0.005
Neoplastic diseases	6 (6)	7 (7)	1.000
Asthma	2 (2)	1 (1)	0.811
HBV infection	6 (2.4)	4 (1.6)	0.751
Neurological disorders	2 (2)	2 (2)	1.000
Others	7 (7)	11 (11)	0.459
Drug consumption			
Yes	44 (44)	24 (24)	0.004
No	56 (56)	76 (76)	
Beta blockers	8 (8)	4 (4)	0.373
ACE inhibitors	6 (6)	8 (8)	0.783
Angiotensin II receptor antagonists (ARBs)	6 (6)	3 (3)	0.498
Thiazide diuretics	6 (6)	2 (2)	0.279
Calcium channel blockers	6 (6)	1 (1)	0.118
Antiplatelets	3 (3)	0 (0)	0.246
Blood thinner	2 (2)	2 (2)	1.000
Statins	9 (9)	0 (0)	0.003⁣^∗∗^
Proton pump inhibitors	9 (9)	0 (0)	0.003⁣^∗∗^
Levothyroxine sodium	11 (11)	2 (2)	0.018
Bisphosphonates	2 (2)	2 (2)	1.000

The significant difference between percentages was measured by Fisher's exact test. ⁣^∗∗^Significance with the Bonferroni correction 0.002 for the systemic diseases. ⁣^∗∗^Significance with the Bonferroni correction 0.003 for the drug consumption. Abbreviations: BMS: burning mouth syndrome; HC: healthy controls; BMI: body mass index.

**Table 3:** Prevalence of COVID-19 infection and covariates, social media use, use across categories of healthcare services, socioeconomic satisfaction, and the subjective feeling of social loneliness during the COVID-19 outbreak.

	BMS	HC	*p* value
COVID-19 infection and covariates	Frequency (%)	Frequency (%)	
COVID-19 infection (yes)	28 (28)	38 (38)	0.176
Quarantine after COVID-19 infection (yes)	28 (28)	36 (36)	0.289
Quarantine for COVID-19 infection of relatives (yes)	23 (23)	23 (23)	1.000
Social media use	Frequency (%)	Frequency (%)	
More time on the Internet after the pandemic (yes)	37 (37)	38 (38)	1.000
Time on Internet (hours)	Median [IQR]	Median [IQR]	
Activities on Internet	2 [1-2.25]	1.25 [1-2.62]	0.628
Instant messaging	2.5 [0.75-3]	3 [2-3]	0.06
Social networking	1 [0-3]	2 [1-3]	0.017
Searching for information	3 [1.75-3]	3 [2-3]	0.146
Entertainment	1 [0-3]	1.5 [0-2]	0.816
Shopping online	0 [0-2]	1 [0-2]	0.156
Booking of travel/social events	0 [0-1]	1 [0-2]	0.018
Financial service blogging	0 [0-1.25]	0 [0-2]	0.148
Debating online/forums	0 [0-0]	0 [0-1]	0.014
Education/learning services	0 [0-2]	1 [0-3]	0.005⁣^∗∗^
Healthcare services	Frequency (%)	Frequency (%)	
Emergency care	24 (24)	20 (20)	0.609
Hospital	23 (23)	13 (13)	0.097
Primary care physician	57 (57)	32 (32)	0.001⁣^∗∗^
Doctor on duty	10 (10)	6 (6)	0.435
Psychiatrist consultation	10 (10)	6 (6)	0.435
Psychologist consultation	15 (15)	8 (8)	0.183
Others	66 (66)	46 (46)	0.007
Level of socioeconomic satisfaction	Median [IQR]	Median [IQR]	
[0-6]	3 [2-4]	3 [2-4]	0.734
Satisfaction in residence	Median [IQR]	Median [IQR]	
[0-6]	5 [3-5]	5 [4-5]	0.314
Social loneliness	Median [IQR]	Median [IQR]	
Number of relatives at home	2 [2-3]	3 [2-4]	0.012⁣^∗∗^
Satisfaction in relationships with relatives (0-6)	4 [1.75-5]	5 [4-5]	<0.001⁣^∗∗^

A significant difference between the percentages was measured by Fisher's exact test. IQR is the interquartile range. The significant difference between medians was measured by the Mann–Whitney *U* test. ⁣^∗^Significance 0.01 < *p* ≤ 0.05. ⁣^∗∗^Significance *p* ≤ 0.01. ⁣^∗∗^Significance with the Bonferroni correction 0.017 for COVID-19 infection and covariates. ⁣^∗∗^Significance with the Bonferroni correction 0.005 for social media use. ⁣^∗∗^Significance with the Bonferroni correction 0.006 for healthcare services. ⁣^∗∗^Significance with the Bonferroni correction 0.025 for social loneliness. Abbreviations: BMS: burning mouth syndrome; HC: healthy controls; BMI: body mass index.

**Table 4:** Analysis of subscores and total score of GHQ, DASS-21, and ISI in 100 BMS patients and in 100 healthy controls.

Clinical parameters	BMS	HC	*p* value
GHQ-12	Median [IQR]	Median [IQR]	
GHQ-1	1 [1-2]	1 [1-1]	0.009
GHQ-2	1 [0-2]	2 [1-2]	0.009
GHQ-3	1 [1-2]	1 [1-1]	0.160
GHQ-4	1 [1-2]	1 [1-1]	0.001⁣^∗∗^
GHQ-5	1 [0-2]	1 [1-2]	0.332
GHQ-6	1 [1-2]	1 [1-2]	0.801
GHQ-7	1 [1-2]	1 [1-2]	0.810
GHQ-8	1 [1-1.25]	1 [1-1]	0.170
GHQ-9	1 [0-2]	1 [1-2]	0.138
GHQ-10	1 [0-2]	1 [1-2]	0.067
GHQ-11	1 [1-2]	1 [1-1]	0.649
GHQ-12	1 [1-2]	1 [1-1]	<0.001⁣^∗∗^
GHQ-12 total score	15 [12-18]	15 [12-17.25]	0.786
DASS-21	Frequency (%)	Frequency (%)	
Stress			
Normal (0-10)	32 (32)	51 (51)	<0.001⁣^∗∗^
Mild (11-18)	35 (35)	37 (37)	
Moderate (19-26)	22 (22)	5 (5)	
Severe (27-34)	7 (7)	7 (7)	
Extremely severe (35-42)	4 (4)	0 (0)	
Anxiety			
Normal (0-6)	45 (45)	69 (69)	0.005
Mild (7-9)	7 (7)	8 (8)	
Moderate (10-14)	27 (27)	14 (14)	
Severe (15-19)	11 (11)	3 (3)	
Extremely severe (20-42)	10 (10)	6 (6)	
Depression			
Normal (0-9)	51 (51)	68 (68)	0.050
Mild (10-12)	14 (14)	13 (13)	
Moderate (13-20)	17 (17)	11 (11)	
Severe (21-27)	8 (8)	6 (6)	
Extremely severe (28-42)	10 (10)	2 (2)	
DASS-21 total score	Median [IQR] 16 [10-24.2]	Median [IQR] 10 [4-17]	<0.001⁣^∗∗^
ISI	Frequency (%)	Frequency (%)	
Normal (0-7)	56 (56)	63 (63)	0.634
Subthreshold insomnia (8-14)	31 (31)	29 (29)	
Moderate insomnia (15-21)	11 (11)	7 (7)	
Severe insomnia (22-28)	2 (2)	1 (1)	
ISI total score	Median [IQR] 6 [3-11]	Median [IQR] 5 [2.75-10]	0.265

A significant difference between percentages was measured by Fisher's exact test. IQR is the interquartile range. The significant difference between medians was measured by the Mann–Whitney *U* test. GHQ and DASS-21: ⁣^∗∗^significance with the Bonferroni correction 0.004. ISI: ⁣^∗∗^significance with the Bonferroni correction 0.006. Abbreviations: BMS: burning mouth syndrome; HC: healthy controls; GHQ: General Health Questionnaire; DASS-21: Depression Anxiety Stress Scales-21; ISI: Insomnia Severity Index.

**Table 5:** Analysis of subscores and total score of ULS-8, MSPSS, and SIDAS in 100 BMS patients and in 100 healthy controls.

Clinical parameters	BMS	HC	*p* value
ULS-8	Median [IQR]	Median [IQR]	
ULS-1	2 [1-3]	2 [1-3]	0.078
ULS-2	2 [1-3]	1 [1-2]	0.144
ULS-3	4 [3-4]	3 [3-4]	0.078
ULS-4	1 [1-2]	1 [1-2]	0.575
ULS-5	1 [1-2]	1 [1-2]	0.170
ULS-6	3 [3-4]	3.5 [3-4]	0.968
ULS-7	1 [1-2]	1 [1-1.25]	0.004⁣^∗∗^
ULS-8	2 [1-3]	1 [1-2]	0.292
ULS-8 total score	16 [14-20.25]	16 [14-17.25]	0.017
MPSS	Median [IQR]	Median [IQR]	
MPSS-1	6 [4-7]	5 [3-7]	0.080
MPSS-2	6 [4.75-7]	5.5 [2-7]	0.044
MPSS3	6 [5-7]	5 [3-7]	0.038
MPSS-4	6 [4-7]	5.5 [4-7]	0.452
MPSS-5	6 [4.75-7]	6 [3-7]	0.146
MPSS-6	4 [2-5]	4 [3-6]	0.176
MPSS-7	4 [2.75-5.25]	4 [2.75-6]	0.353
MPSS-8	6 [4.75-7]	6 [3-7]	0.481
MPSS-9	4 [3-6]	4 [2-6]	0.795
MPSS-10	6 [4.75-7]	6 [3-7]	0.422
MPSS-11	6 [5-7]	6 [3-7]	0.315
MPSS-12	4 [2.75-6]	5 [2.75-6]	0.578
MPSS total score	61 [51-72]	61.5 [40-72]	0.411
Family support	24 [19-28]	22 [14-28]	0.193
Friend support	16 [11-22]	17.5 [11.5-24]	0.493
Significant other support	24 [17.8-28]	22 [11.8-28]	0.133
SIDAS	Median [IQR] 0 [0-0]	Median [IQR] 0 [0-0]	0.335

IQR is the interquartile range. The significant difference between medians was measured by the Mann–Whitney *U* test. ULS-8 and SIDAS: ⁣^∗∗^significance with the Bonferroni correction 0.006. MSPSS: ⁣^∗∗^significance with the Bonferroni correction 0.004. Abbreviations: BMS: burning mouth syndrome; HC: healthy controls; ULS-8: UCLA Loneliness Scale-8; MSPSS: Multidimensional Scale of Perceived Social Support; SIDAS: Suicidal Ideation Attribute Scale.

**Table 6:** Disease onset; type of treatment and number of consultations prior to the diagnosis; type of referral and number of referrals; intensity, quality, and pattern of pain; prevalence of oral symptoms; and location in 100 BMS patients.

Disease onset (months)	Mean ± SD 30 [12-49.5]
Number of doctors consulted prior to diagnosis of BMS	Mean ± SD 2 [1-3]
Referrals	Frequency (%)
Physician	52 (52)
Maxillofacial surgeon	1 (1)
Otolaryngologist	30 (30)
Gastroenterologist	14 (14)
Dentist	82 (82)
Dermatologist	1 (1)
Neurologist	4 (4)
Psychiatrist	1 (1)
Other	8 (8)
Disease onset before pandemic	Frequency (%) 63 (63)
Disease onset after pandemic	Frequency (%) 37(37)
Worsening of disease during pandemic	Frequency (%) 27 (27)
Time of worsening (months)	Median; IQR 12 [5-13]
Treatment with psychotropic drugs	Frequency (%) 62 (62)
Treatment with PEA	Frequency (%) 29 (29)
Increasing dosage after pandemic outbreak (yes)	13 (13)
Pain	Median; IQR
NRS	6 [4-8]
SF-MPQ	6 [1-13.25]
Pattern of symptoms	Frequency (%)
Same in the morning/afternoon/evening	14 (14)
Worse in the afternoon/evening	32 (32)
Worse in the morning	9 (9)
Changing day by day	45 (45)
Present in the night	36 (36)
Oral symptoms	Frequency (%)
Burning	100 (100)
Only burning	11 (11)
Burning+additional symptoms	89 (89)
Intraoral foreign body sensation	21 (21)
Xerostomia	63 (63)
Dysgeusia	42 (42)
Globus pharyngeus	13 (13)
Subjective change in tongue morphology	12 (12)
Subjective change in tongue color	32 (32)
Sialorrhea	17 (17)
Itching	10 (10)
Tingling sensation	24 (24)
Occlusal dysesthesia	17 (17)
Oral dyskinesia	10 (10)
Dysosmia	7 (7)
Subjective halitosis	29 (29)
Location of pain/burning	Frequency (%)
Burning/pain diffuse to entire oral mucosa	40 (30)
Burning/pain localized in one or more sites of oral mucosa	60 (60)
Tongue	80 (80)
Lips	38 (38)
Palate	20 (20)
Gums	38 (38)
Cheeks	38 (38)
Floor of the mouth	19 (19)
Trigone	3 (3)

Abbreviations: BMS: burning mouth syndrome; PEA: palmitoylethanolamide; NRS: Numeric Rating Scale; SF-MPQ: short-form McGill Pain Questionnaire.

**Table 7:** Multiple linear regression model predicting ULS-8 in BMS patients.

Predictors	Model 1		Model 2		Model 3		Model 4		Model 5		Model 6		Model 7		Model 8		Model 9		Model 10		Model 11		Model 12		Model 13		Model 14	
	Beta (SE)	*p* value	Beta (SE)	*p* value	Beta (SE)	*p* value	Beta (SE)	*p* value	Beta (SE)	*p* value	Beta (SE)	*p* value	Beta (SE)	*p* value	Beta (SE)	*p* value	Beta (SE)	*p* value	Beta (SE)	*p* value	Beta (SE)	*p* value	Beta (SE)	*p* value	Beta (SE)	*p* value	Beta (SE)	*p* value
Gender: male	-1.57 (0.97)	0.109	-1.16 (0.98)	0.237	-1.62 (0.98)	0.102	-1.66 (0.99)	0.095	-1.50 (0.98)	0.129	-1.57 (0.98)	0.112	-1.55 (0.98)	0.115	-0.37 (0.81)	0.653	-1.60 (0.97)	0.101	-1.15 (0.95)	0.229	-1.09 (0.87)	0.217	-1.60 0.97	0.103	-1.28 (0.95)	0.184	-0.13 (0.83)	0.874
Age	0.07 (0.03)	0.056	0.05 (0.04)	0.205	0.06 (0.03)	0.072	0.07 (0.03)	0.056	0.07 (0.03)	0.049⁣^∗^	0.07 (0.03)	0.053	0.07 (0.03)	0.050⁣^∗^	0.09 (0.03)	0.002⁣^∗∗^	0.07 (0.03)	0.038⁣^∗^	0.07 (0.03)	0.045⁣^∗^	0.08 (0.03)	0.011⁣^∗^	0.07 (0.03)	0.034⁣^∗^	0.08 (0.03)	0.019⁣^∗^	0.08 (0.03)	0.011⁣^∗^
Education	0.22 (0.10)	0.027⁣^∗^	0.23 (0.10)	0.022⁣^∗^	0.24 (0.10)	0.023⁣^∗^	0.23 (0.10)	0.026⁣^∗^	0.22 (0.10)	0.034⁣^∗^	0.22 (0.10)	0.032⁣^∗^	0.21 (0.10)	0.038⁣^∗^	0.19 (0.08)	0.024⁣^∗^	0.23 (0.10)	0.021⁣^∗^	0.19 (0.10)	0.047⁣^∗^	0.19 (0.09)	0.041⁣^∗^	0.24 (0.10)	0.018⁣^∗^	0.22 (0.10)	0.023⁣^∗^	0.17 (0.09)	0.046⁣^∗^
Marital status: married	-1.14 (0.86)	0.190	-0.77 (0.91)	0.400	-1.06 (0.87)	0.87	-1.09 (0.87)	0.213	-1.16 (0.87)	0.182	-1.17 (0.87)	0.179	-1.22 (0.87)	0.164	-0.54 (0.71)	0.449	-1.13 (0.86)	0.191	-1.37 (0.83)	0.104	0.01 (0.80)	0.991	-0.78 (0.90)	0.389	-0.86 (0.84)	0.312	0.69 (0.85)	0.419
Employment status: employed	-0.45 (0.95)	0.638	0.19 (0.99)	0.851	-0.43 (0.95)	0.649	-0.30 (0.97)	0.758	-0.41 (0.95)	0.664	-0.37 (0.96)	0.699	-0.44 (0.95)	0.641	-0.03 (0.77)	0.974	-0.49 (0.94)	0.600	-0.10 (0.92)	0.918	0.51 (0.87)	0.559	-0.38 (0.94)	0.690	-0.03 (0.93)	0.970	0.75 (0.88)	0.398
Body mass index (BMI)	-0.03 (0.09)	0.684	0.01 (0.09)	0.929	-0.03 (0.09)	0.708	-0.04 (0.09)	0.671	-0.04 (0.09)	0.650	-0.04 (0.09)	0.683	-0.04 (0.09)	0.664	0.00 (0.07)	0.960	-0.04 (0.08)	0.598	-0.03 (0.08)	0.741	-0.01 (0.08)	0.853	-0.03 (0.08)	0.710	-0.03 (0.08)	0.694	0.04 (0.07)	0.571
Number of relatives at home			-0.64 (0.51)	0.218																							-0.30 (0.46)	0.512
Satisfaction in relationships with relatives during the COVID-19 pandemic			-0.35 (0.19)	0.066																							-0.09 (0.16)	0.589
Social media use: more time on the Internet after pandemic (yes)					-0.15 (0.25)	0.537																					-0.13 (0.21)	0.556
COVID-19 infection (yes)							-0.35 (0.91)	0.702																			0.39 (0.80)	0.626
Healthcare service: primary care physician							0.63 (0.81)	0.434																			0.01 (0.69)	0.986
Statins									-0.87 (1.41)	0.537																	0.54 (2.00)	0.787
Proton pump inhibitors											0.75 (1.39)	0.591															-0.34 (1.24)	0.786
Hypercholesterolemia													-0.89 (1.16)	0.443													-0.53 (1.71)	0.756
DASS-21 total															0.19 (0.03)	<0.001⁣^∗∗^											0.17 (0.04)	<0.001⁣^∗∗^
GHQ-12 total																	0.12 (0.08)	0.134									-0.06 (0.07)	0.390
ISI																			0.20 (0.07)	0.004⁣^∗∗^							-0.01 (0.08)	0.918
MSPSS-12 total																					-0.11 (0.02)	<0.001⁣^∗∗^					-0.09 (0.02)	<0.001⁣^∗∗^
NRS																							0.22 (0.16)	0.178			0.16 (0.17)	0.355
SF-MPQ																									0.13 (0.05)	0.012⁣^∗^	-0.05 (0.06)	0.399
*R* ^2^ (%)	7.97	0.024⁣^∗^	10.73	0.011⁣^∗^	7.35	0.036⁣^∗^	6.73	0.052	7.35	0.036⁣^∗^	7.26	0.038⁣^∗^	7.56	0.034⁣^∗^	38.83	<0.001⁣^∗∗^	9.22	0.017⁣^∗^	14.83	0.002⁣^∗∗^	26.52	<0.001⁣^∗∗^	8.79	0.021⁣^∗^	13.22	0.003⁣^∗∗^	42.64	<0.001⁣^∗∗^
*R* ^2^ change (%)			2.77	0.093	-0.614	0.537	-1.23	0.682	-0.61	0.537	-0.71	0.591	-0.40	0.443	30.86	<0.001⁣^∗∗^	1.25	0.134	6.86	0.004⁣^∗∗^	18.55	<0.001⁣^∗∗^	0.83	0.178	5.25	0.012⁣^∗^	34.68	<0.001⁣^∗∗^

SE are the standard errors of the beta estimates. The *p* values were obtained from the hypothesis test on the regression coefficients. ⁣^∗^Moderately significant 0.01 < *p* ≤ 0.05. ⁣^∗∗^Strongly significant *p* value ≤ 0.01.

**Table 8:** Multiple linear regression model predicting ULS-8 in HC.

Predictors	Model 1		Model 2		Model 3		Model 4		Model 5		Model 6		Model 7		Model 8		Model 9		Model 10	
	Beta (SE)	*p* value	Beta (SE)	*p* value	Beta (SE)	*p* value	Beta (SE)	*p* value	Beta (SE)	*p* value	Beta (SE)	*p* value	Beta (SE)	*p* value	Beta (SE)	*p* value	Beta (SE)	*p* value	Beta (SE)	*p* value
Gender: male	-1.66 (0.81)	0.042⁣^∗^	-1.70 (0.79)	0.035⁣^∗^	-1.39 (0.82)	0.094	-1.57 (0.82)	0.057	-1.54 (0.81)	0.059	-1.35 (0.78)	0.088	-1.77 (0.82)	0.034⁣^∗^	-1.73 (0.81)	0.037⁣^∗^	-1.60 (0.81)	0.050⁣^∗^	-0.79 (0.84)	0.348
Age	0.04 (0.06)	0.498	-0.01 (0.06)	0.865	0.04 (0.06)	0.518	0.04 (0.06)	0.568	0.04 (0.06)	0.509	0.06 (0.06)	0.277	0.03 (0.06)	0.664	0.05 (0.06)	0.441	0.03 (0.06)	0.594	-0.01 (0.07)	0.863
Education	0.04 (0.11)	0.741	0.05 (0.11)	0.619	0.00 (0.11)	0.966	0.04 (0.11)	0.690	0.02 (0.11)	0.841	0.08 (0.10)	0.451	0.05 (0.11)	0.655	0.04 (0.11)	0.731	0.05 (0.11)	0.626	0.09 (0.11)	0.434
Marital status: married	-1.89 (0.88)	0.035⁣^∗^	-0.97 (0.97)	0.321	-1.75 (0.88)	0.049⁣^∗^	-1.86 (0.90)	0.041⁣^∗^	-1.99 (0.88)	0.026⁣^∗^	-1.54 (0.85)	0.076	-1.89 (0.88)	0.035⁣^∗^	-1.76 (0.90)	0.055	-1.63 (0.91)	0.076	-0.84 (0.96)	0.383
Employment status: employed	0.61 (0.74)	0.417	0.56 (0.73)	0.445	0.38 (0.75)	0.612	0.70 (0.76)	0.360	0.73 (0.75)	0.329	0.64 (0.72)	0.371	0.59 (0.75)	0.435	0.63 (0.75)	0.404	0.60 (0.74)	0.419	0.71 (0.74)	0.340
Body mass index (BMI)	0.02 (0.10)	0.801	0.08 (0.10)	0.426	-0.01 (0.10)	0.918	0.01 (0.10)	0.907	0.04 (0.10)	0.680	0.01 (0.09)	0.931	0.04 (0.10)	0.686	0.04 (0.10)	0.722	0.04 (0.10)	0.709	0.03 (0.10)	0.770
Number of relatives at home			-0.53 (0.35)	0.129															-0.48 (0.35)	0.172
Satisfaction in relationships with relatives during the COVID-19 pandemic			-0.47 (0.31)	0.133															-0.53 (0.32)	0.102
Social media use: more time on the Internet after pandemic (yes)					0.44 (0.28)	0.112													0.19 (0.28)	0.493
COVID-19 infection (yes)							0.32 (0.78)	0.685											0.29 (0.78)	0.713
Healthcare service: primary care physician							0.68 (0.80)	0.401											0.74 (0.85)	0.388
Hypercholesterolemia									3.44 (2.59)	0.189									4.40 (2.60)	0.094
DASS-21 total											0.11 (0.04)	0.004⁣^∗∗^							0.13 (0.04)	0.004⁣^∗∗^
GHQ-12 total													-0.08 (0.10)	0.446					-0.01 (0.11)	0.944
ISI															0.05 (0.07)	0.503			-0.12 (0.09)	0.180
MSPSS-12 total																	-0.02 (0.02)	0.238	-0.01 (0.02)	0.692
*R* ^2^ (%)	4.88	0.081	8.33	0.029⁣^∗^	6.47	0.051	3.83	0.137	5.64	0.069	12.28	0.005⁣^∗∗^	4.45	0.104	4.31	0.109	5.3	0.078	14.99	0.006⁣^∗∗^
*R* ^2^ change (%)			3.45	0.069	1.59	0.112	-1.04	0.611	0.77	0.189	7.41	0.004⁣^∗∗^	-0.43	0.446	-0.56	0.503	0.42	0.238	10.11	0.033⁣^∗^

SE are the standard errors of the beta estimates. The *p* values were obtained from the hypothesis test on the regression coefficients. ⁣^∗^Moderately significant 0.01 < *p* ≤ 0.05. ⁣^∗∗^Strongly significant *p* value ≤ 0.01.

## Data Availability

The datasets used and/or analyzed during the current study are available from the corresponding author on reasonable request.

## Abbreviations

BMS: Burning mouth syndrome
HC: Healthy controls
GHQ-12: 12-Item General Health Questionnaire
DASS-21: Depression Anxiety Stress Scales-21
ISI: Insomnia Severity Index
ULS-8: Short-form UCLA Loneliness Scale-8
MSPSS: Multidimensional Scale of Perceived Social Support
SIDAS: Suicidal Ideation Attributes Scale.

## Supplementary Materials

Supplementary Materials The questionnaires' design and instructions have been uploaded as Supplementary file 1.
