# Supplementary material for: Loneliness during the COVID-19 Pandemic in Patients with Burning Mouth Syndrome: A Multicentric Case-Control Italian Study
Source: Depress Anxiety. 2023 Nov 8;2023:6666741. doi: 10.1155/2023/6666741 (PMC11921841; doi:10.1155/2023/6666741)
Supplement: Supplementary Materials — The questionnaires' design and instructions have been uploaded as Supplementary file 1. [file 6666741.f1.pdf]

Additional File 1

Section 1 – ***Socio-demographic form (SSD)***

1. Current residence (region): _________________________
2. Current residence (city): ________________________
3. Current residence (province): _______________________
4. Gender: ☐ Male ☐ Female ☐ Other
5. Age: _____________
6. Weight (kg): ______________
7. Height (cm): ___________
8. City of birth: ____________________________
9. Province of birth: ________________________
10. Country of birth: _________________________
11. Marital status:
    ☐ Married/partner ☐ Separated/divorced ☐ Widower ☐ Single
12. Educational level:
    ☐ Primary School ☐ Junior High School ☐ High School ☐ Professional School ☐ University
    ☐ Other: _________________________
13. Currently student: ☐ Yes ☐ No
14. Currently worker: ☐ Yes ☐ No
15. After the pandemic outbreak did you lose your job? ☐ Yes ☐No
16. Job:
    ☐ Househusband/housewife ☐ Unemployed ☐ Employed ☐ Self-employed
    ☐ Healthcare provider ☐ Retired for age ☐ Retired for illness ☐ Other: _____________
17. After the pandemic outbreak, how much are you gratified concerning your financial situation?
    ☐ It could not be worse ☐ Unsatisfied ☐ Quite unsatisfied ☐ Halfway
    ☐ Quite satisfied ☐ Satisfied ☐ It could not be better
18. How many people do you live with? (Including you) _____________________
19. After the pandemic outbreak, how much are you satisfied by the people you live with? Or how much are you satisfied by living alone (if you live by yourself)?
    ☐ It could not be worse ☐ Unsatisfied ☐ Quite unsatisfied ☐ Halfway
    ☐ Quite satisfied ☐ Satisfied ☐ It could not be better

20. After the pandemic outbreak, how much are you satisfied with the place where you live?
    ☐ It could not be worse ☐ Unsatisfied ☐ Quite unsatisfied ☐ Halfway
    ☐ Quite satisfied ☐ Satisfied ☐ It could not be better

21. After the pandemic outbreak, do you spend more time on the Internet? ☐ Yes ☐ No
22. For which activities do you use the Internet?

	Never	Rarely	Sometimes	Often	Always
Instant messaging	☐	☐	☐	☐	☐
Social network	☐	☐	☐	☐	☐
Search for information	☐	☐	☐	☐	☐
Entertainment	☐	☐	☐	☐	☐
Shopping on-line	☐	☐	☐	☐	☐
Booking of travel/social events	☐	☐	☐	☐	☐
Financial services	☐	☐	☐	☐	☐
Blog/debating on-line/forum	☐	☐	☐	☐	☐
Education/learning services	☐	☐	☐	☐	☐

23. How much time do you usually spend on the Internet (hours)? ______________________

24. Do you suffer from any physical disorder? ☐ Yes ☐ No

25. Do you suffer from any psychiatric disorder? ☐ Yes ☐ No

Section 2 – *Burning Mouth Syndrome Questionnaire*

1. How long have you been suffering from Burning Mouth Syndrome (months)?
   ____

2. How many doctors have you been visited by before being diagnosed with Burning Mouth Syndrome? ______________________________

3. Which clinician(s) have you been visited by?
   ☐ Dentist ☐ Otolaryngologist ☐ General doctor
   ☐ Other (specify): ____

4. Has the Burning Mouth Syndrome always been steady since its first onset? ☐ Yes ☐ No

5. Did you already suffer from Burning Mouth Syndrome before the pandemic outbreak? ☐ Yes ☐ No
   If yes, has the Burning Mouth Syndrome exacerbated after the pandemic outbreak? ☐ Yes ☐ No
   If yes, how long has it been getting worse? ________________________________

6. Has the Burning Mouth Syndrome currently reduced since its worsening or is it steady?
   ☐ Reduced ☐ Steady ☐ I have not had any worsening

7. Which areas of the oral cavity are involved by the symptoms?
   ☐ Tongue ☐ Gums ☐ Lips ☐ Cheeks ☐ Floor of mouth ☐ Palate
   ☐ Other (specify): ____

8. Please, read carefully each adjective below describing your pain. Place a “X” mark in the column that corresponds to your level of pain. Do this for <u>each</u> word.

	None (0)	Mild (1)	Moderate (2)	Severe (3)
1. Throbbing	☐	☐	☐	☐

2. Shooting	☐	☐	☐	☐
3. Stabbing	☐	☐	☐	☐
4. Sharp	☐	☐	☐	☐
5. Cramping	☐	☐	☐	☐
6. Gnawing	☐	☐	☐	☐
7. Hot-burning	☐	☐	☐	☐
8. Aching	☐	☐	☐	☐
9. Heavy	☐	☐	☐	☐
10. Tender	☐	☐	☐	☐
11. Splitting	☐	☐	☐	☐
12. Tiring-Exhausting	☐	☐	☐	☐
13. Sickening	☐	☐	☐	☐
14. Fearful	☐	☐	☐	☐
15. Cruel-Punishing	☐	☐	☐	☐

9. In addition to burning, do any of the following symptoms occur?
   ☐ Dysgeusia
   ☐ Xerostomia
   ☐ Sialorrhea
   ☐ Itching
   ☐ Intraoral Foreign Body Sensation
   ☐ Subjective Halitosis
   ☐ Tingling Sensation
   ☐ Change of Tongue Morphology
   ☐ Change of Tongue Colour
   ☐ Dysosmia
   ☐ Oral Dyskinesia
   ☐ Occlusal Dysesthesia
   ☐ None of the above
   ☐ Other (specify): ____

10. Are the symptoms steady throughout the day?
    ☐ Steady ☐ Milder in the morning and increased in the evening
    ☐ Fluctuating throughout the day ☐ Other:____

11. Do the symptoms occur during the night? ☐Yes ☐ No

12. Have you ever taken drugs to reduce Burning Mouth Syndrome symptoms?
    ☐ Pharmacological therapy ☐ OTC drugs/supplements ☐ Nothing

13. How intense is the burning sensation?
    Consider “10” as the most intense pain you can imagine.
    ☐ 1 ☐ 2 ☐ 3 ☐ 4 ☐ 5 ☐ 6 ☐ 7 ☐ 8 ☐ 9 ☐ 10

Section 3 - *Covid-19 Questions*

1. Did you get the COVID-19 infection? ☐ Yes ☐ No

2. Have you been isolated after contracting the infection? ☐ Yes ☐ No

3. Have you been isolated after getting in contact with someone positive? ☐ Yes ☐ No

Section 4 – *GHQ-12*

In the last year:

	More than usual	As usual	Less than usual	Much less than usual
1. Have you been able to concentrate well on what you were doing?	☐	☐	☐	☐
2. Have you lost much sleep over worry?	☐	☐	☐	☐
3. Have you felt that you are playing a useful part in things?	☐	☐	☐	☐
4. Have you felt capable of making decisions?	☐	☐	☐	☐
5. Have you felt constantly overwhelmed and stressed?	☐	☐	☐	☐
6. Have you felt like you cannot overcome difficulties?	☐	☐	☐	☐
7. Did you enjoy your day-to-day activities and get your free time?	☐	☐	☐	☐
8. Did you adequately cope with your problems?	☐	☐	☐	☐
9. Have you felt unhappy or depressed?	☐	☐	☐	☐
10. Have you lost confidence in yourself?	☐	☐	☐	☐
11. Have you thought that you are a person worthless?	☐	☐	☐	☐
12. Do you feel reasonably happy considering all the circumstances?	☐	☐	☐	☐

Section 5 – *DASS-21*

Please, read each sentence and mark how often the described situation has occurred in the last year. Consider that there are neither correct nor incorrect answers. Do not spend too much time on any statement, often the first answer is the most accurate. Thank you for your precious willingness and collaboration.

	Did not apply to me at all	Applied to me to some degree	Applied to me to a considerable degree	Applied to me most of the time
1. I felt a lot of nervous tension and I found it hard to wind down	☐	☐	☐	☐
2. I was aware of dryness of my mouth	☐	☐	☐	☐
3. I could not seem to experience any positive feeling at all	☐	☐	☐	☐
4. I experienced breathing difficulty (e.g., excessively rapid breathing, breathlessness in the absence of physical exertion)	☐	☐	☐	☐
5. I found it difficult to work up the initiative to do things	☐	☐	☐	☐
6. I tended to over-react to situations	☐	☐	☐	☐
7. I experienced trembling (e.g., in the hands)	☐	☐	☐	☐
8. I felt that I was using a lot of nervous energy	☐	☐	☐	☐
9. I was worried about situations in which I might panic and make fool of myself	☐	☐	☐	☐
10. I felt that I had nothing to look forward to	☐	☐	☐	☐
11. I felt stressed	☐	☐	☐	☐
12. I found it difficult to relax	☐	☐	☐	☐
13. I felt down-hearted and blue	☐	☐	☐	☐
14. I felt intolerant of anything that kept me from getting on with what I was doing	☐	☐	☐	☐
15. I felt I was close to panic	☐	☐	☐	☐

16. I was unable to become enthusiastic about anything	☐	☐	☐	☐
17. I felt I wasn't worth much as a person	☐	☐	☐	☐
18. I felt that I was rather touchy	☐	☐	☐	☐
19. I was aware of the action of my heart in the absence of physical exertion (e.g., sense of heart rate increase, heart missing a beat)	☐	☐	☐	☐
20. I felt scared without any good reason	☐	☐	☐	☐
21. I felt that life was meaningless	☐	☐	☐	☐

Section 6 – *OCI-R*

The following statements refer to experiences that many people deal with in their everyday lives. Mark the box that best describes how much that experience has distressed or bothered you during the last year.
Consider that there are no correct nor incorrect answers. Do not spend too much time on any statement, often the first answer is the most accurate. Thank you.

	**Not at all**	**A little**	**Moderately**	**A lot**	**Extremely**
1. I have saved up so many things that they get in the way	☐	☐	☐	☐	☐
2. I check things more often than necessary	☐	☐	☐	☐	☐
3. I get upset if objects are not arranged properly	☐	☐	☐	☐	☐
4. I feel compelled to count while I am doing things	☐	☐	☐	☐	☐
5. I find difficult to touch an object when I know it has been touched by strangers or certain people	☐	☐	☐	☐	☐
6. I find difficult to control my own thoughts	☐	☐	☐	☐	☐
7. I collect things I don't need	☐	☐	☐	☐	☐
8. I repeatedly check doors, windows, drawers, etc.	☐	☐	☐	☐	☐
9. I get upset if others change the way I have arranged things	☐	☐	☐	☐	☐
10. I feel I have to repeat certain numbers	☐	☐	☐	☐	☐
11. I sometimes have to wash or clean myself simply because I feel contaminated	☐	☐	☐	☐	☐
12. I am upset by unpleasant thoughts that come into my mind against my will	☐	☐	☐	☐	☐
13. I avoid throwing things away because I am afraid I might need them later	☐	☐	☐	☐	☐

14. I repeatedly check gas and water taps and light switches after turning them off	☐	☐	☐	☐	☐
15. I need things to be arranged in a particular way	☐	☐	☐	☐	☐
16. I feel that there are good and bad numbers	☐	☐	☐	☐	☐
17. I wash my hands more often and longer than necessary	☐	☐	☐	☐	☐
18. I frequently get nasty thoughts and have difficulty in getting rid of them	☐	☐	☐	☐	☐

Section 7 – *ISI*

1. Please, rate the current (i.e., last year) severity of your insomnia problems.

	**None**	**Mild**	**Moderate**	**Severe**	**Very severe**
Difficulty falling asleep	☐	☐	☐	☐	☐
Difficulty staying asleep	☐	☐	☐	☐	☐
Problem waking up too early	☐	☐	☐	☐	☐

2. How satisfied/unsatisfied are you with your current sleep pattern?
☐ Very satisfied ☐ Satisfied ☐ Neutral ☐ Unsatisfied ☐ Very unsatisfied

3.

	**Not at all**	**A little**	**Somewhat**	**Much**	**Very much**
To what extent do you consider your sleep problem to interfere with your daily functioning (e.g. daytime fatigue, ability to function at work/daily chores, concentration, memory, mood, etc.)?	☐	☐	☐	☐	☐
How noticeable to others do you think your sleeping problem is in terms of impairing the quality of your life?	☐	☐	☐	☐	☐
How worried/distressed are you about your current sleep problem?	☐	☐	☐	☐	☐

Section 8 – *NSESS*

Please, mark how often did you feel like described in the following sentences during the last year.

	**Not at all**	**Rarely**	**Sometimes**	**Often**	**Always**
1. Having "flashbacks", that is, you suddenly acted or felt as if a stressful experience from the past was happening all over again (for example, you reexperienced parts of a stressful experience by seeing, hearing, smelling, or physically feeling parts of the experience)?	☐	☐	☐	☐	☐
2. Feeling very emotionally upset when something reminded you a stressful experience?	☐	☐	☐	☐	☐
3. Feeling detached from reality, your body or your memories?	☐	☐	☐	☐	☐

4. Trying to avoid thoughts, feelings, or physical sensations that reminded you of a stressful experience?	☐	☐	☐	☐	☐
5. Being "super alert", on guard, or constantly on the lookout for danger?	☐	☐	☐	☐	☐
6. Feeling jumpy or easily startled when you hear an unexpected noise?	☐	☐	☐	☐	☐
7. Being extremely irritable or angry to the point where you yelled at other people, got into fights, or destroyed things?	☐	☐	☐	☐	☐

Section 9 – *SIDAS*

Please, answer to the following question choosing a number from 0 (never) to 9 (always).

1. In the last year, how often have you thought about suicide?

☐ 0 ☐ 1 ☐ 2 ☐ 3 ☐ 4 ☐ 5 ☐ 6 ☐ 7 ☐ 8 ☐ 9

Section 10 – *IES-R*

Below there is a list of difficulties people sometimes must face after stressful life events. Please, read each item and then indicate how distressing each difficulty has been for you during the last year with respect to the event. How much were you distressed or bothered by these difficulties?

	**Not at all**	**A little bit**	**Moderately**	**Quite a bit**	**Extremely**
1. I thought about the stressful event when I didn't mean to	☐	☐	☐	☐	☐
2. I tried not to think about it	☐	☐	☐	☐	☐
3. I was aware that I still had a lot of feelings about it, but I didn't deal with them	☐	☐	☐	☐	☐
4. I had trouble concentrating	☐	☐	☐	☐	☐
5. I felt watchful and on-guard regarding environment and people	☐	☐	☐	☐	☐
6. Other things kept making me think about it	☐	☐	☐	☐	☐

Section 11 – ULS-8

Please, read each item and then indicate how often you have felt like described in each sentence in the last year.

	**Never**	**Rarely**	**Sometimes**	**Always**
1. I lack companionship	☐	☐	☐	☐
2. There is no one I can turn to	☐	☐	☐	☐
3. I am an outgoing person	☐	☐	☐	☐
4. I feel left out	☐	☐	☐	☐
5. I feel in isolation from others	☐	☐	☐	☐
6. I can find companionship when I want it	☐	☐	☐	☐
7. I am unhappy being so withdrawn	☐	☐	☐	☐

8. People are around me but not with me	☐	☐	☐	☐

Section 12 - *Brief Cope*

Please, try to answer each question like if it is detached from others. Choose the answer that is the most truthful for you. There are no correct nor incorrect answers, choose it without thinking about what "most people" would say or do. Read the statements and indicate what you do when you face a stressful event.

	**I usually do not do this at all**	**I usually do this a little bit**	**I usually do this a medium amount**	**I usually do this a lot**
1. I turn to work or other activities to take my mind off things	☐	☐	☐	☐
2. I concentrate my efforts on doing something about the situation I'm in	☐	☐	☐	☐
3. I say to myself "this isn't real"	☐	☐	☐	☐
4. I use alcohol or other drugs to make myself feel better	☐	☐	☐	☐
5. I get emotional support from others	☐	☐	☐	☐
6. I give up trying to deal with it	☐	☐	☐	☐
7. I take action to try to make the situation better	☐	☐	☐	☐
8. I refuse to believe that it has happened	☐	☐	☐	☐
9. I say things to let my unpleasant feelings escape	☐	☐	☐	☐
10. I get help and advice from other people	☐	☐	☐	☐
11. I use alcohol or other drugs to help me get through it	☐	☐	☐	☐
12. I try to see it in a different light, to make it seem more positive	☐	☐	☐	☐
13. I have been criticizing myself	☐	☐	☐	☐
14. I try to come up with a strategy about what to do	☐	☐	☐	☐
15. I get comfort and understanding from someone	☐	☐	☐	☐
16. I give up the attempt to cope	☐	☐	☐	☐
17. I look for something good in what is happening	☐	☐	☐	☐
18. I make jokes about it	☐	☐	☐	☐
19. I do something to think about it less, such as going to movies, watching TV, reading, daydreaming, sleeping, or shopping	☐	☐	☐	☐
20. I accept the reality of the fact that it has happened	☐	☐	☐	☐
21. I express my negative feelings	☐	☐	☐	☐
22. I try to find comfort in my religion or spiritual beliefs	☐	☐	☐	☐

23. I try to get advice or help from other people about what I should do	☐	☐	☐	☐
24. I learn to live with it	☐	☐	☐	☐
25. I think hard about what steps to take	☐	☐	☐	☐
26. I blame myself for things that happened	☐	☐	☐	☐
27. I pray or meditate	☐	☐	☐	☐
28. I make fun of the situation	☐	☐	☐	☐

Section 13 –*PTGI – SF*

Please, read each statement and indicate the life change level occurred after a traumatic event, in a scale from 0 (no change) to 5 (very important life change).

	**0**	**1**	**2**	**3**	**4**	**5**
1. Change of life values	☐	☐	☐	☐	☐	☐
2. Greater appreciation for the value of my own life	☐	☐	☐	☐	☐	☐
3. Deep spirituality	☐	☐	☐	☐	☐	☐
4. Establishing a new life path	☐	☐	☐	☐	☐	☐
5. Greater sense of closeness with others	☐	☐	☐	☐	☐	☐
6. I know better that I can handle difficulties	☐	☐	☐	☐	☐	☐
7. I appreciate more every new day	☐	☐	☐	☐	☐	☐
8. I have a stronger religious faith	☐	☐	☐	☐	☐	☐
9. I discovered that I’m stronger than I thought I was	☐	☐	☐	☐	☐	☐
10. I learned a great deal about how wonderful people are	☐	☐	☐	☐	☐	☐

Section 14 – *Connor-Davidson Resilience Scale – short version*

Please, read each item and then indicate how you have been feeling in the last year in a scale that goes from 0 (rarely true) to 5 (about always true).

	**0**	**1**	**2**	**3**	**4**	**5**
1. I am able to adapt when changes occur	☐	☐	☐	☐	☐	☐
2. I can deal with whatever comes	☐	☐	☐	☐	☐	☐
3. I try to see humous side of problems	☐	☐	☐	☐	☐	☐
4. Coping with stress can strengthen me	☐	☐	☐	☐	☐	☐
5. I tend to bounce back after illness or hardship	☐	☐	☐	☐	☐	☐
6. I can achieve goals despite obstacles	☐	☐	☐	☐	☐	☐
7. I can stay focused under pressure	☐	☐	☐	☐	☐	☐
8. I am not easily discouraged by failure	☐	☐	☐	☐	☐	☐
9. I think of myself as strong person	☐	☐	☐	☐	☐	☐
10. I can handle unpleasant feelings	☐	☐	☐	☐	☐	☐

Section 15 – *Use of medical services and surgical procedures form*

Since the pandemic outbreak:

1. Have you been taking medicines? ☐ Yes ☐ No
   If yes, for which condition? ____

2. If you have been taking medicines, have you increased their dosage since the pandemic outbreak?
   ☐ Yes ☐ No ☐ I do not take medicines

3. Have you ever accessed the emergency room? ☐ Yes ☐ No
   If yes, why? ____

4. Have you been hospitalized? ☐ Yes ☐ No
   If yes, why? ____

5. Have you been visited by your general practitioner? ☐ Yes ☐ No
   If yes, why? ____

6. Have you been visited by a medical guard? ☐ Yes ☐ No
   If yes, why? ____

7. Have you done a psychiatric consult? ☐ Yes ☐ No
   If yes, why? ____

8. Have you done other medical consults? ☐ Yes ☐ No
   If yes, which and why? ____

9. Have you done psychological interviews? ☐ Yes ☐ No
   If yes, how often? ____
   Why? ____

Section 16 – ***Multidimensional Scale of Perceived Social Support***
Please, for each statement indicate a number from 1 (absolutely false) to 7 (absolutely true).

	1	2	3	4	5	6	7
1. There is a special person who is around when I am in need	☐	☐	☐	☐	☐	☐	☐
2. There is a special person with whom I can share my joys and sorrows	☐	☐	☐	☐	☐	☐	☐
3. My family really tries to help me	☐	☐	☐	☐	☐	☐	☐
4. I get the emotional help and support I need from my family	☐	☐	☐	☐	☐	☐	☐
5. I have a special person who is a real source of comfort to me	☐	☐	☐	☐	☐	☐	☐
6. My friends really try to help me	☐	☐	☐	☐	☐	☐	☐
7. I can count on my friends when things go wrong	☐	☐	☐	☐	☐	☐	☐
8. I can talk about my problems with my family	☐	☐	☐	☐	☐	☐	☐
9. I have friends with whom I can share my joys and sorrows	☐	☐	☐	☐	☐	☐	☐
10. There is a special person in my life who cares about my feelings	☐	☐	☐	☐	☐	☐	☐
11. My family is willing to help me make decisions	☐	☐	☐	☐	☐	☐	☐
12. I can talk about my problems with my friends	☐	☐	☐	☐	☐	☐	☐
